# The expression of cystathionine gamma-lyase is regulated by estrogen receptor alpha in human osteoblasts

**DOI:** 10.18632/oncotarget.21514

**Published:** 2017-10-04

**Authors:** Elisabetta Lambertini, Letizia Penolazzi, Marco Angelozzi, Francesco Grassi, Laura Gambari, Gina Lisignoli, Pasquale De Bonis, Michele Cavallo, Roberta Piva

**Affiliations:** ^1^ Department of Biomedical and Specialty Surgical Sciences, University of Ferrara, Ferrara, Italy; ^2^ Ramses Laboratory, Rizzoli Orthopedic Institute, Bologna, Italy; ^3^ Laboratory of Immunorheumatology and Tissue Regeneration, Rizzoli Orthopedic Institute, Bologna, Italy; ^4^ Department of Neurosurgery, S. Anna University Hospital, Ferrara, Italy

**Keywords:** cystathionine gamma-lyase, H_2_S, osteoblasts, bone, estrogen receptor alpha

## Abstract

Hydrogen sulfide (H_2_S), generated in the osteoblasts predominantly via cystathionine-γ-lyase (CSE), is bone protective. Previous studies suggested that the onset of bone loss due to estrogen deficiency is associated to decreased levels of H_2_S and blunted gene expression of CSE. However, there are still a lot of unknowns on how H_2_S levels influence bone cells function. The present study aims to explore the mechanisms by which estrogen may regulate CSE expression, in particular the role of estrogen receptor alpha (ERα) in human osteoblasts (hOBs). Vertebral lamina derived hOBs were characterized and then assessed for CSE expression by western blot analysis in the presence or absence of ERα overexpression. Bioinformatic analysis, luciferase reporter assay and ChIP assay were performed to investigate ERα recruitment and activity on hCSE gene promoter.

Three putative half Estrogen Responsive Elements (EREs) were identified in the hCSE core promoter and were found to participate in the ERα – mediated positive regulation of CSE expression. All osteoblast samples responded to ERα over-expression increasing the levels of CSE protein in a comparable manner. Notably, the ERα recruitment on the regulatory regions of the CSE promoter occurred predominantly in female hOBs than in male hOBs. The obtained results suggest that CSE/H_2_S system is in relation with estrogen signaling in bone in a gender specific manner.

## INTRODUCTION

Cystathionine gamma-lyase (CSE) is the predominant hydrogen sulfide (H_2_S)-producing enzyme in mammalian cells although other systems including that supported by cystathionine β-synthase (CBS) exist [1].

The interest in the CSE enzyme and its regulation is growing, due to the recently described functions of H_2_S including the S-sulphydration of various target proteins [2]. H_2_S is a highly diffusible gasotransmitter, that influences cellular and organ functions by different mechanisms, including cross-interactions with other signaling pathways not yet fully elucidated [3]. The antioxidant and anti-inflammatory properties together with the cytoprotective effects of H_2_S seem to be so critical that abnormal H_2_S metabolism has been linked to several human pathologies, including autoimmune diseases, Alzheimer’s, hypertension, coronary heart disease, atherosclerosis, cataracts, pancreatitis, type 1 diabetes, osteoporosis and rheumatoid arthritis [3–6]. It is therefore urgent to understand the tissue-dependent control of its production.

In the recent years, many factors have been discovered regulating CSE expression and activity in mouse and human, at transcriptional, post-transcriptional and post-translational levels [7]. Several compounds, including butyrate, specific hormones, calcium, streptozotocin (STZ), nitric oxide (NO), lipopolysaccharides (LPS), ovalbumin (OVA), pyridoxal-5′-phosphate (PLP), vitamin D, S-propargyl-cysteine, and various H_2_S donors, have been described as positive modulators of CSE activity and H_2_S production [7–10]. In contrast, glucose, heme oxygenase-1/carbon monoxide (HO-1/CO) system, and various CSE inhibitors suppress CSE activity and, consequently, H_2_S production [11, 12].

For what concerns pathophysiological conditions of the bone, CSE-H_2_S was found as a dominant H_2_S generation system in osteoblasts promoting osteoblast activity by the pathway of the calcium channel [13] and osteogenic master regulator Runx2 [14]. In particular, sulfhydrated Runx2 enhanced its transactivation and increased osteoblast differentiation and maturation, thereby promoting bone healing [14]. CSE expression is up-regulated during *in vitro* osteogenic process and is positively correlated with the mineral matrix deposition [15]. Conversely, decreased serum levels of H_2_S together a decrease of CSE expression was recently found in mice with osteoporosis caused by estrogen deficiency [16].

However, whether the H_2_S biosynthesis system in bone context is directly regulated by estrogen via its receptor action has not been clearly examined. To test the hypothesis that estrogen protects from bone loss [17] through CSE up-regulation in osteoblasts, we investigated whether the estrogen receptor alpha (ERα) directly participates in the regulation of CSE gene promoter activity and in the modulation of CSE expression in human male and female osteoblasts.

## RESULTS

### Characterization of osteoblasts from human vertebral lamina bone

The cells used in the experiments were obtained from human vertebral lamina bone fragments and are defined hereafter as human primary osteoblasts (hOBs) based on the below described characteristics.

**Figure 1:**
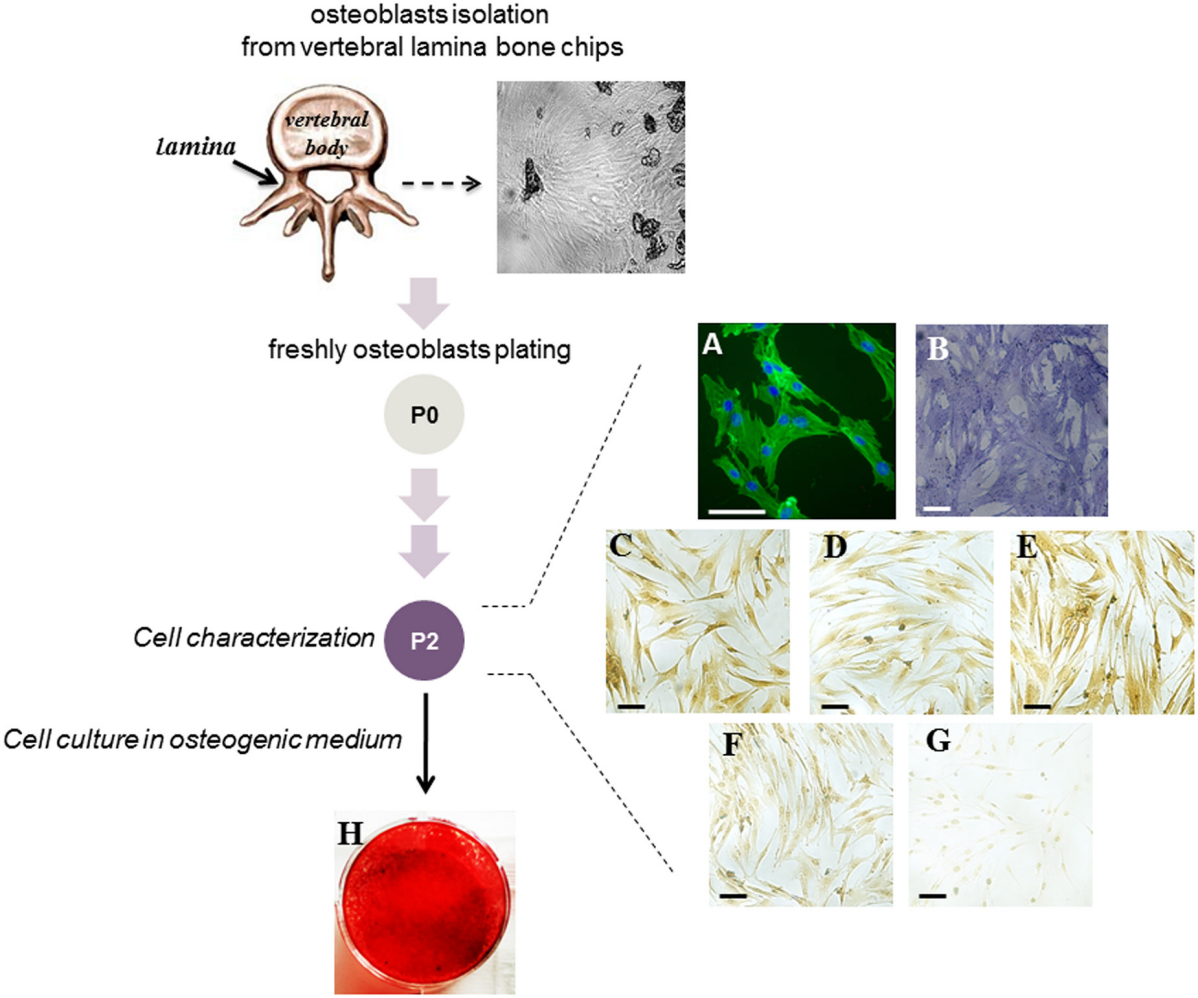
Characterization of the cells obtained from vertebral lamina bone chips A scheme of the experimental approach is reported. A representative image of bone fragments obtained from vertebral lamina bone is shown. The cells (hOBs) were analyzed at P2 passage of culture. Cell-associated green fluorescence reveals actin cytoskeleton (Alexa Fluor 488-conjugated phalloidin); blue fluorescence indicates cell nuclei (DAPI DNA staining) **(A)**. The analysis of ALP activity by specific staining **(B)** and the expression of RUNX2 **(C)**, OPN **(D)**, Collagen type I **(E)**, ERα **(F)** and ERβ **(G)** by immunocytochemistry is reported. The ability of cells to deposit mineralized matrix was evaluated after 10 days of culture in osteogenic medium by Alizarin Red staining **(H)**. Bars correspond to 50 μm.

Cells migrating out of the bone chips were grown until confluent, trypsinized, counted, and passaged. Analysis for the expression of bone markers was performed on cells from passage 2 (P2), cultured in non-osteogenic medium. The morphology of adherent cells was analyzed by fluorescence microscopy after staining of cytoskeletal F-actin filaments. As shown in Figure 1A, the cells exhibited an abundant thin F-actin filament meshwork typical for mature osteoblasts [18]. Mature osteoblast phenotype was verified by analyzing the expression of the osteogenic markers including alkaline phosphatase (ALP), Runx2 transcription factor belonging to Runt-related factors family, osteopontin (OPN), and Collagen type I (COL1). As shown in Figure 1B–1E, the cells spontaneously expressed ALP which is among the first functional genes expressed in the process of mineralization [19], Runx2 which is essential in regulating the expression of the principal osteoblast-specific genes [20], OPN which is one of the abundant non-collagenous proteins in bone matrix [21], and COL1, the most abundant protein in bone [22].

Moreover, cells were positively immunostained for estrogen receptor alpha (ERα) in both cytoplasmic and nucler compartment, but showed only a faint nuclear positivity for estrogen receptor beta (ERβ) (Figure 1F, 1G). The ability of cells to deposit mineralized matrix was evaluated in osteogenic cell culture medium; interestingly, after few days the cells formed the typical osteogenic clusters, and were strongly stained by Alizarin Red already at day 10 of culture (Figure 1H).

Based on the measured parameters, the cell population from vertebral lamina includes bona fide mature osteoblasts.

### ERα affects CSE expression

**Figure 2:**
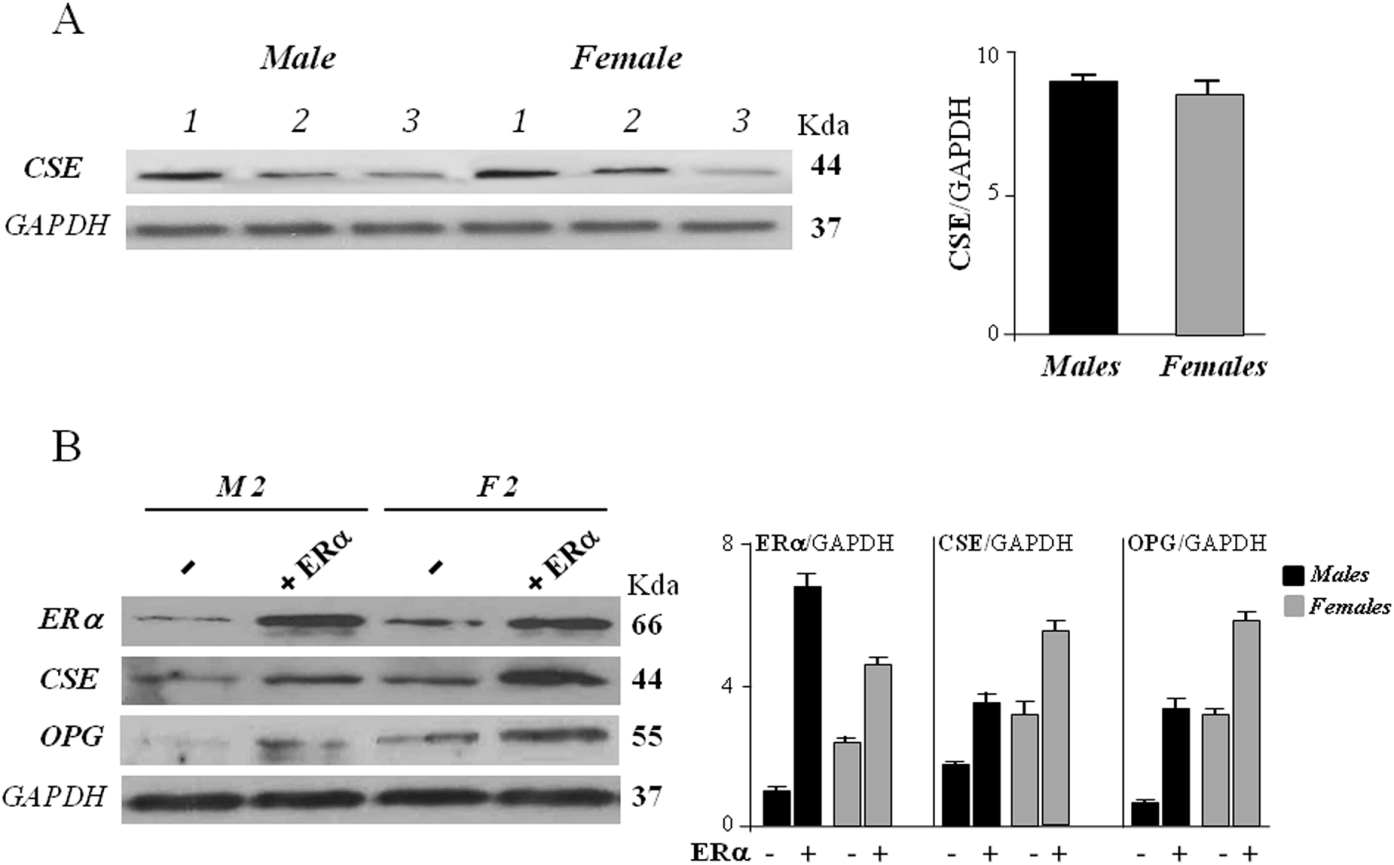
CSE protein expression in hOBs from male (M) and female (F) donors **(A)** The cells were harvested after 72 hours of culture and subjected to Western blot analysis for CSE expression. Bar graphs show the densitometric analysis of all samples analyzed. GAPDH expression was used as the internal control to evaluate total protein of samples loaded, data were expressed as ratio of CSE in respect to GAPDH and presented as mean + standard deviation, SD (n=3 for male group, n=3 for female group). **(B)** The cells were exposed to ERα overexpression together 10 nM 17β-estradiol for 48 hours (+ ERα) or remained untreated (-) and subjected to Western blot analysis for ERα, CSE and OPG expression. Representative Western blot is reported (sample 2, M2, for male group, and sample 2, F2, for female group). Bar graphs show the densitometric analysis of all samples analyzed. GAPDH expression was used as the internal control to evaluate total protein of samples loaded, data were expressed as ratio of ERα, CSE and OPG in respect to GAPDH and presented as mean + standard deviation, SD (n=3 for male group, n=3 for female group).

We then evaluated the CSE expression level on each sample distinguishing male from female. As reported in Figure 2A, all samples regardless of gender, expressed CSE at comparable levels.

When hOBs were transfected with ERα expression vector and exposed to 10 nM 17-β-estradiol for 48 hours, a remarkable increase of CSE protein expression was observed in both female and male cells (Figure 2B). These data suggest that ERα exerts a positive effect on the entire CSE transcriptional unit and its action may be so strong that it predominates over that of other regulatory factors and mechanisms.

Increased expression levels of ERα and CSE resulted also in a significant upregulation of osteoprotegerin (OPG) in all samples analyzed (Figure 2B). Several studies have indicated that estrogen stimulates OPG expression in osteoblast cells at transcriptional level through ERα [23]. A recent paper on human periodontal ligament cells (hPDLCs), proposed that H_2_S could promote osteogenic differentiation by regulating OPG, since increased OPG expression was found after exogenous H_2_S treatment [24]. To the best of our knowledge, the present study is the first reporting a positive correlation between CSE and OPG expression in human osteoblasts, suggesting that estrogen-mediated OPG increase may be due both to a direct action of estrogen/ERα complex on OPG gene promoter, and to an increase of endogenous H_2_S production.

### Potential estrogen responsive elements in the hCSE core promoter

**Figure 3:**
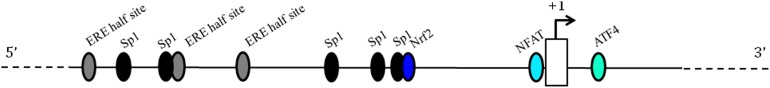
Schematic representation of the 5’-flanking region of the human CSE gene The transcription factors up to now identified as regulators of CSE transcription are reported as ovals. Putative Estrogen Receptor binding sites are indicated as ERE half sites. Nrf2 (nuclear factor E2-related factor 2, involved in the antioxidant cell response), ATF4 (activating transcription factor 4, binds the cAMP response element), NFAT (the calcium-regulated nuclear factor of activated T cells) and Sp1 (specificity protein 1, an important component of the eukaryotic cellular transcriptional machinery) binding sites are indicated.

The bioinformatic search for potential ER binding sites (Estrogen Responsive Elements, EREs) (Patch 1.0 and AliBaba 2.1 public software) in the hCSE core promoter (-592/+139) did not return any canonical consensus cis-elements. However, three putative half EREs were found at positions -564, -414, and -300, together with few other validated transcription factor binding sites (Figure 3). Since it has been described that ERα can regulate the transcription of genes that contain ERE half-sites in their promoters [25], we focused our attention on these three half EREs.

### ERα regulates hCSE promoter activity

To determine whether the three half EREs in the hCSE core promoter could be responsible for mediating its activity, and to analyze the functional involvement of ERα in the regulation of hCSE expression, we generated a luciferase reporter construct, cloning the hCSE core promoter upstream of firefly luciferase gene (LUC) in the pGL3-Basic vector (pGL3-731).

**Figure 4:**
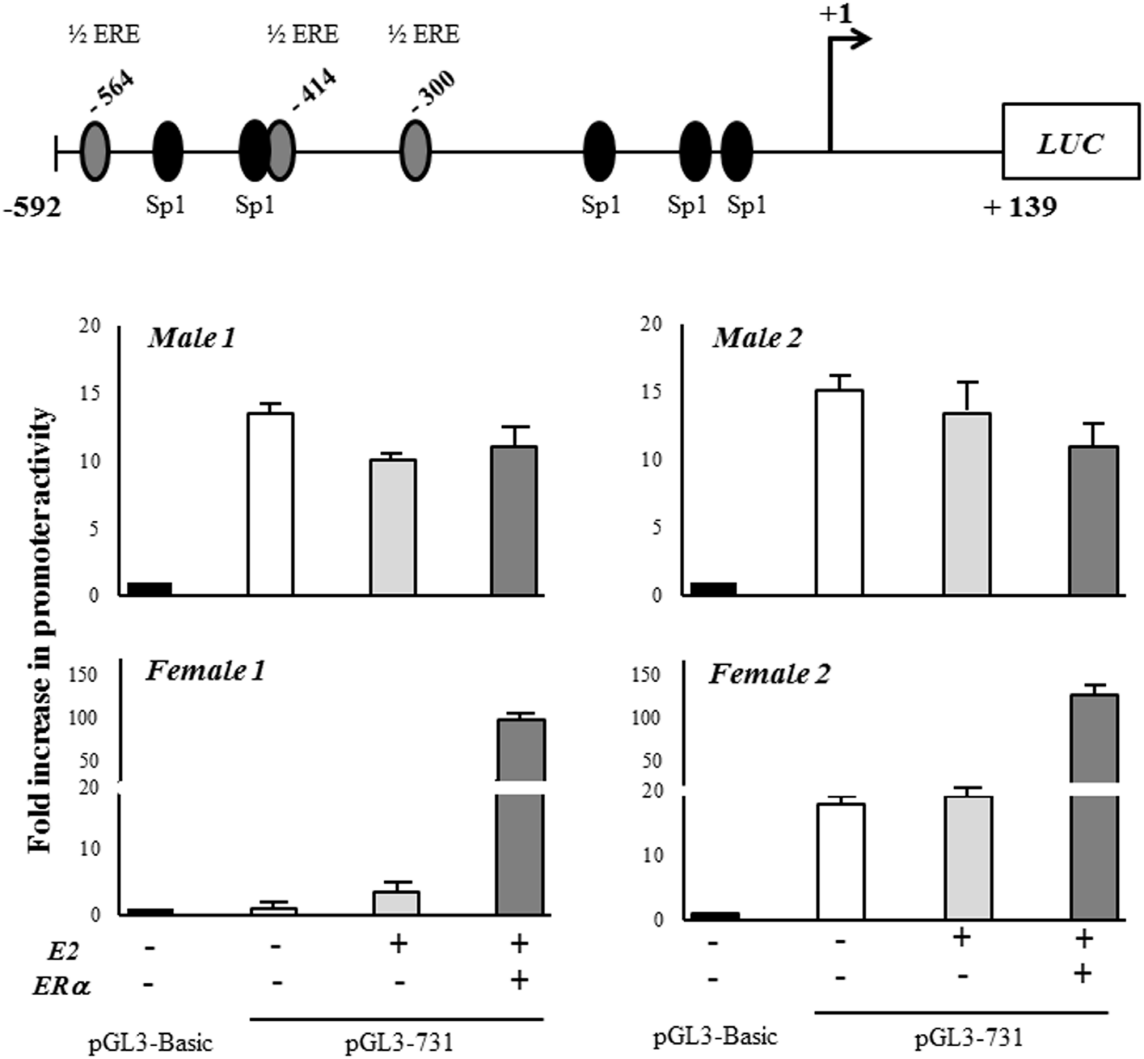
CSE gene promoter activity The core promoter of CSE gene (-592/+139) was cloned into the upstream of firefly luciferase gene (LUC) in the pGL3-Basic vector (pGL3-731). The transfection was performed in hOB samples from two male and two female donors in absence (-) or in presence (+) of ERα expression vector together 10 nM 17-β-estradiol (E2) for 48 hours. The transfection efficiency was normalized by measuring the total protein in the extracts. The LUC output was normalized against the promoterless pGL3-Basic vector. Results are expressed as mean + SD.

The analysis showed that the construct displayed an activity about 15 times higher than pGL3-Basic control vector in three out four analyzed samples, suggesting that it would contain some important cis-acting elements (Figure 4). When hOBs were co-transfected with pSG5HEO hERα expression vector in the presence of 10 nM 17-β-estradiol, they were differently susceptible to ERα overexpression. Specifically, ERα overexpression deeply enhanced CSE promoter activity in female hOBs but not in male hOBs. On the contrary, exogenous Sp1 overexpression (data not shown) as well as estrogen exposure alone did not significantly alter the promoter activity in hOBs.

These results suggest that gender specific co-regulators molecules may exist, and that the susceptibility to regulation by estrogen in hOBs depends on pathways that are different in male and female.

### ERα is *in vivo* recruited to hCSE promoter

**Figure 5:**
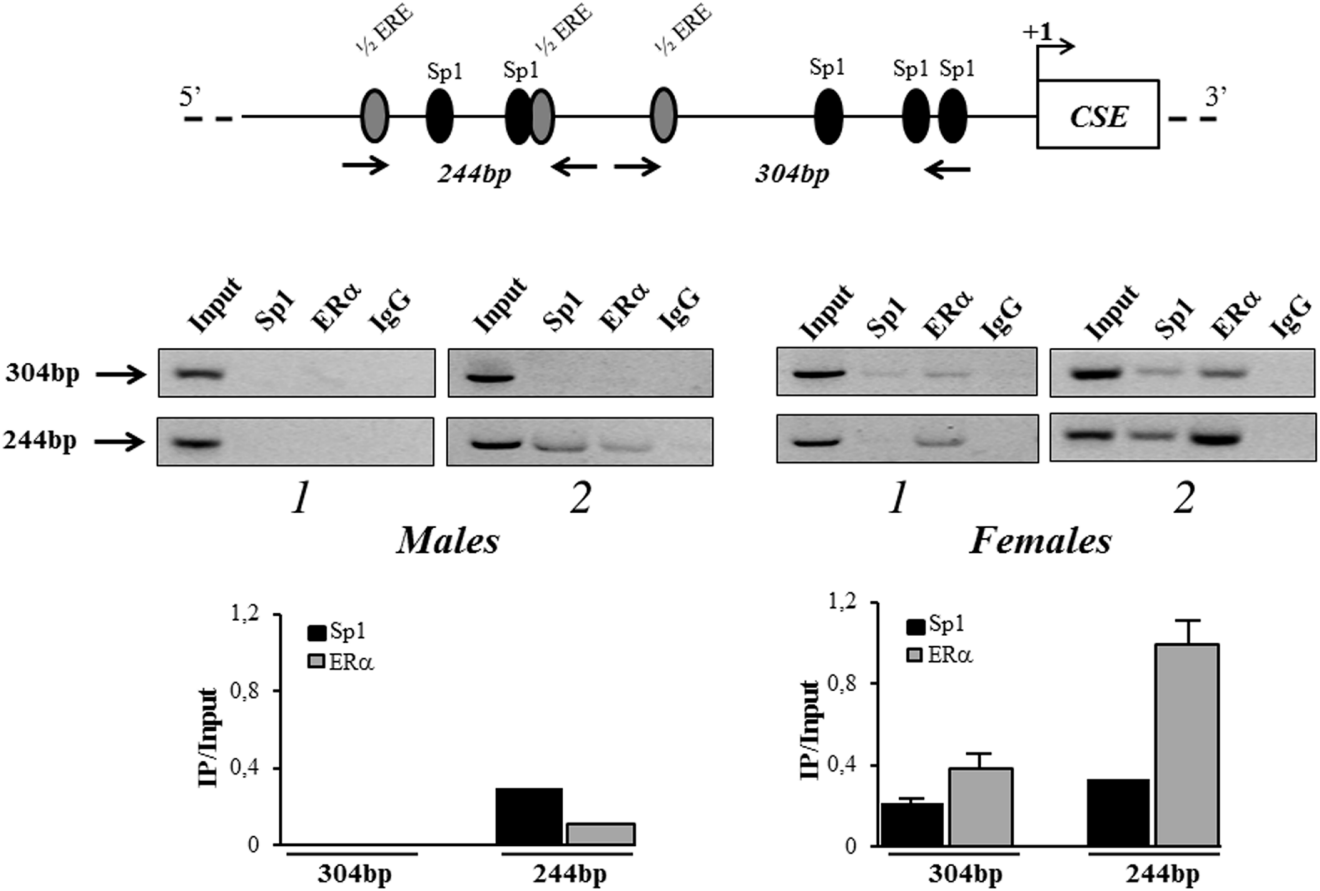
*in vivo* recruitment of ERα to CSE gene promoter Protein–DNA complexes were *in vivo* formaldehyde cross-linked in hOBs from male and female donors and subjected to Chromatin immunoprecipitation (ChIP) analysis. Chromatin fragments were subjected to immunoprecipitation with antibodies against hERα or hSp1. After cross-link reversal, the co-immunoprecipitated DNA was detected by semi-quantitative PCR using the primer pairs spanning the proximal 304 bp region (-348/-44) and 244 bp region (-592/-348) on the CSE core promoter. Aliquots of chromatin taken before immunoprecipitation were used as input positive controls, whereas the negative control was the IgG immunoprecipitation. For each sample, levels of recruited ERα or Sp1 in the immunoprecipitates were determined by densitometry of the PCR fragments relative to Input control. Data are presented as mean + SD.

The *in vivo* binding of ERα to hCSE gene promoter was next investigated. For this purpose ChIP assay was performed by using specific primers that amplify the identified potential ERα binding sites in the 304 bp region (-348/-44) and 244 bp region (-592/-348) (Figure 5). This analysis revealed, for the first time, the ERα recruitment to the proximal region of hCSE promoter. Interestingly, in support of our hypothesis, ERα was stronger recruited in female hOBs than in male hOBs. The Sp1 binding previously described [8] was confirmed in three out four samples analyzed.

## DISCUSSION

H_2_S is a highly reactive molecule and, being a gas, it cannot be stored in vesicles or other biological compartments. As a consequence, the transcriptional regulation of H_2_S-producing enzymes is a key limiting step in the control of H_2_S bioavailability [2]. H_2_S is predominately and primarily produced by three enzymatic pathways, which include cystathionine β-syntase (CBS), cystathionine γ-lyase (CSE) and 3-mercaptopyruvate sulfur transferase (3-MST) [1–5]. CSE is the most studied being the predominant H_2_S-producing enzyme in mammalian cells [2].

Many recent papers attribute an important role to H_2_S levels in the bone microenvironment [13–16, 26], and suggest a therapeutic potential of H_2_S against bone loss caused by estrogen deficiency [16]. It has been demonstrated that H_2_S levels decrease in aging [27] and H_2_S-deficient mice displayed an osteoporotic phenotype, which could be rescued by treatment with donor of H_2_S [13]. H_2_S protects osteoblastic cells from H_2_O_2_-induced cell injury [28], mitigates bone loss in rat with spinal cord injury [29], and has beneficial effect on bone formation in patients affected by periodontitis [30]. H_2_S is also able to regulate osteoblastogenesis and is relevant for the acquisition and preservation of bone mass controlling bone marrow stromal cells function by regulating Ca^2+^ influx [13, 16].

These studies, together the evidence that bone loss induced by estrogen deficiency in ovariectomized mice may be prevented by pharmacological restoration of normal H_2_S levels [16], relate H_2_S biosynthesis with estrogen signaling, however without providing any evidence of direct molecular interactions with enzymes producing H_2_S in bone.

In the present study, for the first time, we demonstrated that estrogen receptor alpha (ERα) directly participates in the regulation of CSE promoter activity and CSE protein expression in human osteoblasts. Importantly, all osteoblast samples responded to ERα over-expression increasing the levels of CSE protein in a comparable manner. However, the ERα recruitment on the regulatory regions of the CSE promoter occurred predominantly in female hOBs. This is in agreement with many evidences showing that ER signaling is not a core osteogenic pathway by itself, but regulates an array of osteogenic modulators including, in this case, potential gender specific co-regulators that provide correct H_2_S levels. Therefore, the present paper open the way to new studies focusing on H_2_S target proteins [31] that may also act as ERα partners. Hypothetical candidates may be sulfhydrated transcription factors or chromatin remodeling enzymes capable to differently modulate antioxidant, antiapoptotic and cytoprotective programs since they are differently sensitive to a gender specific microenvironment.

This hypothesis linked to the wide modulation of the H_2_S signaling comes from a series of observations that demonstrate how many factors have been discovered to regulate CSE expression and activity at multiple levels, including transcriptional, post-transcriptional and post-translational levels. It is worth noting that up to now a small number of transcription factors, including Sp1, Nrf2, ATF4, Elk1 and NFAT, has been demonstrated to regulate CSE transcription through direct or indirect binding with CSE promoter [8, 32, 10, 33, 34]. miR-21 and miR-22 are reported to suppress but PI3K and TNF-α stimulate CSE transcription by targeting Sp1 gene [35–37]. A recent paper investigating H_2_S-microRNA crosstalk in cardiovascular diseases and estrogenic cardioprotection against oxidative stress, demonstrated that estrogen activates ERα, which binds to Sp1 increasing CSE production in murine cardiomyocytes [9]. ERα also inhibits miR-22 which may also inhibit ERα, providing a secondary pathway for reducing CSE expression [35]. H_2_O_2_ and PDGF-BB induce CSE transcription by targeting Nrf2 [38].

Moreover, CSE/H_2_S system can be activated by compounds such as butyrate, specific hormones, calcium, streptozotocin, nitric oxide, lipopolysaccharide, ovalbumin, pyridoxal-5′-phosphate, vitamin D, S-propargyl-cysteine, and various H_2_S donors [7–10]. In contrast, glucose, HO-1/CO system, and various CSE inhibitors are shown to suppress CSE/H_2_S system [11, 12].

Even if there are still a lot of unknowns on how H_2_S levels influence cell function, however all these evidences suggest that crosstalk between H_2_S and other signaling pathways may vary in a context-dependent manner and may have different roles in different disease conditions. Therefore, in relation to future perspectives for H_2_S as a therapeutic agent, it is urgent to clarify how H_2_S levels are regulated in the different tissues.

Considering that many of the estrogen regulated genes in osteoblasts are dependent on membrane ERα signaling [39], it remains to be explored how cross-talk between membrane and nuclear ERα signaling may influence CSE/H_2_S system.

Again, it is noteworthy another aspect regarding the importance of CSE/H_2_S system in the bone context, namely in bone tissue engineering. It has in fact been recently demonstrated that cells exposed to H_2_S have potential applications in regenerative medicine, since modulation of H_2_S metabolism may serve as a therapeutic approach to promote the viability of transplanted mesenchymal stem cells (MSCs) and facilitate MSC-based regeneration [40, 41].

In conclusion and in line with all evidences here reported, we speculated that factors influencing CSE expression and H_2_S signaling (including estrogens themselves) become important for the development of strategies for bone protection and healing, in particular in women. Therefore, the understanding of gender-related H_2_S biosynthesis might be an interesting challenge for the gender medicine field, to evaluate the perspectives of pharmacological enhancement or inhibition of CSE activity, as a strategy for new drug design in the bone female pathological context [42].

## MATERIALS AND METHODS

### Isolation and culture of human osteoblasts

Human bone fragments (explants) from vertebral lamina, discarded from spinal surgery were obtained from 6 patients (3 males and 3 females) using research protocol approved by Ethics Committee of the University of Ferrara and S. Anna Hospital (protocol approved on November 17, 2016). Patients’ ages were between 53 and 73 years (mean age 63 years). Bone chips were dissected into smaller pieces and plated in T-25 culture flasks (Sarstedt, Nümbrecht, Germany) in 50% DMEM high-glucose/50% DMEM F-12/ 20% Fetal Calf Serum (FCS) (Euroclone S.p.A., Milan, Italy), supplemented with 1 mM L-Glutamine, antibiotics (penicillin 100 μg/mL and streptomycin 10 μg/mL) (Sigma Aldrich, St. Louis, USA). Upon detection of a cell colony from the bone fragments, the cells were expanded until confluent (passage zero, P0). Then the cells were harvested after treatment with 0.05 % trypsin ethylenediamine tetraacetic acid (EDTA; Sigma-Aldrich), washed, counted by hemocytometric analysis, and used for further *in vitro* experiments. During the culture period, cells were incubated at 37°C in a humidified atmosphere of 5% CO_2_ and the medium was changed every 3 or 4 days. hOBs (P2) were characterized for the presence of alkaline phosphatase activity (ALP Leukocyte kit cat. no. 86R; Sigma-Aldrich); for analysis of F-actin organization, cells were fixed with 4% PFA (paraformaldehyde) for 30 min, permeabilized with 0.1% Triton X-100 for 10 min, and stained with Alexa Fluor-488 phalloidin (cat.no. sc-363791, Santa Cruz Biotechnology, Dallas, USA) in a phosphate-buffered saline (PBS) solution. For osteogenic induction, hOBs were cultured up to 21 days in osteogenic medium consisting in DMEM high-glucose 10% FCS, supplemented with 10 mM β-glycerophosphate, 10^−7^ M dexamethasone and 100 μM ascorbate (Sigma-Aldrich). The extent of mineralized matrix in the plate was determined by Alizarin Red S staining (ARS; cat. no. A5533, Sigma-Aldrich). The cells were then fixed in 70% methanol for 1h at room temperature, washed with PBS, stained with 40 mM ARS (pH 4.2) for 10 min at room temperature, washed five times with deionized water, and incubated in PBS for 15 min to eliminate non-specific staining. The stained matrix was observed at different magnifications using a Leitz microscope.

### Immunocytochemistry

Immunocytochemistry was performer by using the ImmPRESS (cat.no. MP-7500, Vector Labs, Burlingame, USA). hOBs were fixed in cold 100% methanol and permeabilized with 0.2% (v/v) Triton X-100 in TBS (Tris-Buffered Saline) 1X. After blocking with serum, polyclonal antibodies for OPN (LF-123), COL1A1 (H-197 #sc-28657), Runx2 (M-70 #sc-10758), ERα (G-20 # sc-544), or ERβ (H-150 #sc-8974) (rabbit antihuman, 1:200 dilution, Santa Cruz Biotechnology), were added and incubated overnight (4°C). Cells were then incubated in Vectastain ABC (Vector Labs) reagent 30 min and stained with DAB solution (cat.no. SK-4105, Vector Labs). After washing, cells were mounted in glycerol and observed with the use of the Nikon Eclipse 50i optical microscope.

### ERα overexpression

For transfection experiments, the cells were plated in 6-well plates and maintained in phenol-red free DMEM containing 10% charcoal stripped FCS, and were transiently transfected with 2.5 μg of pSG5HEO (the wild-type human ERα expression vector) using LipofectamineTM 2000 transfection reagent (Thermo Fisher Scientific, Waltham, USA) in the presence of 10 nM 17-β-estradiol (Sigma-Aldrich). 48 h post transfection, cells were harvested and lysed for Western blot analysis.

### Western blots

Details of the Western blot analysis were described previously [43]. Briefly, 20 μg of each sample were electrophoresed on a 12% SDS-polyacrylamide gel. The proteins were then transferred onto PVDF membrane (Millipore, Billerica, USA). After blocking with PBS-0.05% Tween 20 and 5% BSA (Sigma-Aldrich), the membrane was probed with the following antibodies: CSE (mouse anti-human, 1:1000 dilution, cat.no. H00001491-M03, Abnova, Taipei, Taiwan), ERα (rabbit anti-human, 1:1000 dilution, cat.no. sc-543, Santa Cruz Biotechnology), OPG (rabbit anti-human, 1:200 dilution, cat.no. sc-11383, Santa Cruz Biotechnology) and GAPDH (mouse anti-human, 1:5000 dilution, cat.no. MA1-16783, Thermo Fischer Scientific). After washing with PBS-Tween 20, the membranes were incubated with peroxidase-conjugated anti-mouse or anti-rabbit secondary antibody (Dako, Glostrup, Denmark). Immunocomplexes were detected using Immobilon Western Chemiluminescent HRP Substrate (Millipore). GAPDH was used to confirm equal protein loading. Densitometric analysis was performed by ImageJ software (NIH, USA, public domain available at: http://rsb.info.nih.gov/nih-image).

### *In silico* analysis of CSE promoter

The prediction of estrogen responsive elements and Sp1 transcription factor binding sites in the CSE 5’-flanking region spanning +139 to -592 was performed using Patch 1.0 and AliBaba 2.1 public software. The bioinformatic analysis of CSE promoter region failed to identify canonical ERE sites but found three ERE half-sites and confirmed five Sp1 consensus sites already previously described [8].

### Plasmid construct

A 731 bp fragment containing the 5’-flanking region of the human CSE gene (-592 to +139) was generated by PCR using human genomic DNA of human HEK-293 cells as template. The PCR product was digested with *XhoI* and *HindIII* (Promega, Madison, USA) and subsequently cloned into the promoterless pGL3 Basic vector containing a firefly luciferase cDNA (Promega), generating the pGL3-731 construct.

### Transfection and luciferase reporter assay

For each experiment, 4 × 10^4^ hOBs were seeded into 24-well plates in 500 μL phenol red-free DMEM containing 10% charcoal stripped FCS. The cells were cultured for 48 hours and then co-transfected with 0.5 μg of the reporter construct and 0.25 μg of pSG5HEO using Lipofectamine 2000 Reagent, according to the manufacturer's instructions. 24 hours after transfection, the medium was replaced with fresh phenol red-free medium and stimulated, where required, with 10 nM 17-β-estradiol for another 24 hours. Cells were then washed once with PBS and lysed in 120 μL of 1X passive lysis buffer (Promega). The luciferase activity was measured using the Luciferase Assay System (Promega) in a GloMax 20/20 single tube Luminometer (Promega) and corrected for total protein content.

For fold change, the luciferase output was normalized against the promoterless pGL3-Basic vector, arbitrarily defined as 1. For each hOB sample all transfections were performed in triplicate, and data were presented as mean values with standard deviation.

### Chromatin immunoprecipitation (ChIP)

ChIP was conducted as previously described [44]. ChIP assay was performed using a ChIP Assay Kit (cat.no. 17-295 Upstate, USA) following the manufacturer's instructions. After cross linking, chromatin was fixed with 1% formaldehyde at 37°C for 10 min, cells were washed with cold PBS, scraped, collected on ice, lysed and sonicated. An equal amount of chromatin was immunoprecipitated at 4°C overnight with 5 μg of the following ChIP grade antibodies: ERα #sc-543X, Sp1 #sc-14027, or non-specific IgG #sc-2027X (Santa Cruz Biotechnology). Immunoprecipitated products were collected after incubation with Protein A-agarose beads. The beads were washed, and the bound chromatin was eluted in ChIP eluition buffer. The samples were incubated at 65°C overnight to reverse the cross linking. The proteins were then digested with Proteinase K for 1 h at 45°C and DNA was purified in 50 μL of Tris–EDTA with a PCR purification kit (Qiagen, Hilden, Germany) according to the manufacturer's instructions. The DNA precipitates and Input (2% of total chromatin used for the immunoprecipitation) were further subjected to semi-quantitative PCR using specific primers pairs spanned the proximal 304 bp region (-348/-44) and 244 bp region (-592/-348) on the CSE core promoter. PCR products were analyzed by agarose gel electrophoresis and visualized by UV light apparatus. Densitometric analysis was performed by ImageJ software.

### Statistical analysis

Data are presented as mean values with standard deviation from at least three replicates for each sample.

## Abbreviations

CBS: cystathionine β-synthase
CSE: cystathionine γ-lyase
ChIP: chromatin immunoprecipitation
GAPDH: glyceraldehyde 3-phosphate dehydrogenase
ER: estrogen receptor
EREs: estrogen responsive elements
hOBs: human osteoblasts
H_2_S: hydrogen sulfide
Sp1: specificity protein 1
